# A Nonsense Variant in Hephaestin Like 1 (*HEPHL1*) Is Responsible for Congenital Hypotrichosis in Belted Galloway Cattle

**DOI:** 10.3390/genes12050643

**Published:** 2021-04-26

**Authors:** Thibaud Kuca, Brandy M. Marron, Joana G. P. Jacinto, Julia M. Paris, Christian Gerspach, Jonathan E. Beever, Cord Drögemüller

**Affiliations:** 1 Department of Farm Animals, Vetsuisse-Faculty, University of Zurich, 8057 Zurich, Switzerland; thibaud.kuca@uzh.ch (T.K.); cgerspach@vetclinics.uzh.ch (C.G.); 2 Laboratory of Molecular Genetics, Department of Animal Sciences, University of Illinois at Urbana-Champaign, Urbana, IL 61801, USA; bmarron@dacc.edu (B.M.M.); jbeever@utk.edu (J.E.B.); 3 Department of Veterinary Medical Sciences, University of Bologna, 40064 Ozzano Emilia, Italy; joana.goncalves2@studio.unibo.it; 4 Institute of Genetics, Vetsuisse Faculty, University of Bern, 3012 Bern, Switzerland; julia-1991@hotmail.com; 5 UTIA Genomics Center for the Advancement of Agriculture, Institute of Agriculture, University of Tennessee, Knoxville, TN 37996, USA

**Keywords:** *Bos taurus*, hypotrichosis simplex, hair, development, dermatology, monogenic, genodermatosis

## Abstract

Genodermatosis such as hair disorders mostly follow a monogenic mode of inheritance. Congenital hypotrichosis (HY) belong to this group of disorders and is characterized by abnormally reduced hair since birth. The purpose of this study was to characterize the clinical phenotype of a breed-specific non-syndromic form of HY in Belted Galloway cattle and to identify the causative genetic variant for this recessive disorder. An affected calf born in Switzerland presented with multiple small to large areas of alopecia on the limbs and on the dorsal part of the head, neck, and back. A genome-wide association study using Swiss and US Belted Galloway cattle encompassing 12 cases and 61 controls revealed an association signal on chromosome 29. Homozygosity mapping in a subset of cases refined the HY locus to a 1.5 Mb critical interval and subsequent Sanger sequencing of protein-coding exons of positional candidate genes revealed a stop gain variant in the *HEPHL1* gene that encodes a multi-copper ferroxidase protein so-called hephaestin like 1 (c.1684A>T; p.Lys562*). A perfect concordance between the homozygous presence of this most likely pathogenic loss-of-function variant and the HY phenotype was found. Genotyping of more than 700 purebred Swiss and US Belted Galloway cattle showed the global spread of the mutation. This study provides a molecular test that will permit the avoidance of risk matings by systematic genotyping of relevant breeding animals. This rare recessive *HEPHL1*-related form of hypotrichosis provides a novel large animal model for similar human conditions. The results have been incorporated in the Online Mendelian Inheritance in Animals (OMIA) database (OMIA 002230-9913).

## 1. Introduction

Heritable hair disorders frequently follow a monogenic mode of inheritance, e.g., congenital hypotrichosis (HY), and they belong to a group of human diseases, which are classified into syndromic and non-syndromic forms [1]. Generally, HY is characterized by a paucity or the presence of a less than normal amount of hair and abnormal hair follicles and shafts, which are thin and atrophic, and the lesion extension and the affected areas of the body can be very variable [2,3]. Therefore, HY includes a clinically-, pathologically- and heritably-heterogeneous group of hair disorders. At present, 14 subtypes and 12 different associated genes are encompassed in the classification of human non-syndromic HY (OMIM PS605389). These disorders in humans follow mostly a dominant inheritance and are associated with causative variants in eight different genes (*EPS8L3*, *SNRPE*, *CDSN*, *HR*, *KRT71*, *KRT74*, *RPL21*, *APCDD1*) [2,4,5,6,7,8,9,10]. Whereas, autosomal recessive HY is related to mutations in only four different genes (*LIPH*, *LPAR6*, *DSG4*, *LSS*) [11,12,13,14].

Several forms of non-syndromic HY have been reported in many animal species (OMIA 000540), including American mink [15], cats [16,17], dogs [18], horses [19], macaques [20], meadow voles [21], Mongolian gerbils [22], golden hamsters [23], guinea pigs [24], pigs [25], sheep [26] and cattle [27]. Mutations causing forms of HY in animals have been identified in known candidate genes for HY such as *HR* and *KRT71* [16,17], or novel genes such as *TSR2*, *SGK3*, and *SP6* [18,19,27] discovered in HY-affected domestic animals. This highlights the potential of studying inherited conditions in such species to assign a role or function to previously uncharacterized genes or to add additional functions to known genes in regard to hair development.

In Hereford cattle, non-syndromic recessively inherited HY has been reported and it is characterized by the partial or complete absence of hair at birth that later becomes “fuzzy or kinky” in appearance [28]. To the best of our knowledge, in Belted Galloway cattle no forms of HY have been previously reported. Therefore, this study aimed to characterize the clinical phenotype of HY in Belted Galloway cattle and to identify the causative genetic variant associated with the disorder.

## 2. Materials and Methods

### 2.1. Animals and Samples

The clinical investigation was performed in 2020 at the University of Zurich using one affected red Belted Galloway calf from a farm in Switzerland. The original genetic investigation that led to the identification of the pathogenic variant associated with the disease was performed in 2011 at the University of Illinois.

The final mapping population of this study consisted of twelve HY-suspected Belted Galloway calves, eleven of them born in the USA and reported by five different farmers between 2009 to 2011 (cases HYG_0046, HYG_0047, HYG_0048, HYG_0049, HYG_0050, HYG_0051, HYG_0053, HYG26, HYG33, HYG34, HYG-54) and a single HY-suspected calf born in Switzerland (case RM3402). As controls, a total of 61 phenotypically normal Belted Galloway cattle including 21 animals that were collected from US farms and 40 Swiss cattle were used. Eighteen out of the 21 US animals were suspected carriers for HY.

Finally, a population cohort consisting of 156 and 541 apparently normal cattle of the respective Swiss and US purebred Belted Galloway populations were used to determine the absence/presence and frequency of the detected *HEPHL1* variant in the breed.

DNA was isolated from EDTA-blood and semen samples using a simple salting out procedure [29].

### 2.2. Clinicopathological Investigations

An 11-day-old male calf, weighing 43 kg, was admitted to the Clinic for Ruminants of the University of Zurich for a history of progressive hair loss beginning three days after birth. Hair loss was first noticed to affect the head and subsequently the limbs. The calf was born without assistance, weighing 37 kg. The dam was apparently healthy and had previously given birth to three healthy calves. The calf was reported to have nursed and gained weight normally since birth. The affected animal was clinically examined. Furthermore, blood was collected for a complete blood count (CBC), plasma biochemical analysis, and venous blood gas (VBG) analysis. A skin biopsy sample was also collected from the left ear and assayed for bovine viral diarrhea virus (BVDV) by an independent laboratory using a commercially available ELISA kit (IDEXX BVDV Ag/Serum Plus, IDEXX Switzerland AG, Liebefeld-Bern, Switzerland) according to the manufacturer’s instructions.

### 2.3. Genetic Investigations

#### 2.3.1. SNP Genotyping and GWAS

Twelve HY-suspected affected animals (cases) and 61 normal Belted Galloway cattle (controls) were genotyped using the BovineSNP50 v1 Beadchip (Illumina, San Diego, CA, USA). The generated SNP data was prepared for a genome-wide association study (GWAS) and subsequent haplotype analysis using PLINK v1.9 [30]. GWAS using 39,014 informative SNP markers was performed using a linear mixed model while adjusting for population stratification as implemented in GEMMA v0.98 [31] and the significance threshold was estimated by Bonferroni correction. Manhattan and Q–Q plots of the corrected p-values were generated in R environment v3.6.0 [32], using the qqman package [33]. Haplotypes around the significantly associated locus were constructed using fastPHASE [34].

#### 2.3.2. Candidate Gene Analysis

Three genes (*CEP295*, *C29H11orf54*, *HEPHL1*) were selected as candidates based on known function in hair and location relative to the homozygous region identified in the HY-affected calves on chromosome 29. Exon annotation based on computational methods was manually validated using mRNA sequences from NCBI and the software SPIDEY [35]. Primers were designed as described above to amplify protein-coding exons in 1kb fragments (Table S1). Subsequent Sanger sequencing was performed on an ABI3730xl capillary sequencer and sequence assemblies viewed and analyzed for polymorphisms using Codon Code Aligner (Codon Code Corporation).

#### 2.3.3. Targeted Genotyping

A diagnostic PCR and subsequent Sanger sequencing as described above were used to validate and genotype the variant in further animals. Therefore, the region containing the stop gain variant in *HEPHL1* (Chr29: g. 721234T>A) was amplified using the following primers: 5′- TGAAAGTGTCAGCCCAACAG-3′ (forward primer) and 5′- TCGATTTCGAGAGCACTGAG-3′ (reverse primer).

#### 2.3.4. Occurrence of the HEPHL1 Variant in the 1000 Bull Genomes Project Cohort

The most likely pathogenic stop gain variant in *HEPHL1* was searched for its occurrence in a global control cohort of 4110 genomes of a variety of breeds (1000 Bull Genomes Project run 8; www.1000bullgenomes.com accessed on 4 April 2021) [36].

#### 2.3.5. Sequence Accessions

All references to the bovine *HEPHL1* gene correspond to the NCBI accessions NM_001192511.2 (*HEPHL1* mRNA), NP_001179440.1 (HEPHL1 protein), NC_037356.1 (ARS-UCD1.2 assembly, chromosome 29). For the protein structure of HEPHL1, the Uniprot accession F1N752 was used.

## 3. Results

### 3.1. Clinicopathological Findings

On clinical examination, the Swiss calf was found to be clinically healthy except for the skin abnormalities. Particular clinical examination of the cardiovascular, respiratory, genitourinary, musculoskeletal, and nervous systems showed no abnormalities. Moreover, no abnormalities in dentition were noticed as previously seen in cattle affected by ectodermal dysplasia characterized by sparse hair and abnormal teeth [37].

**Figure 1:**
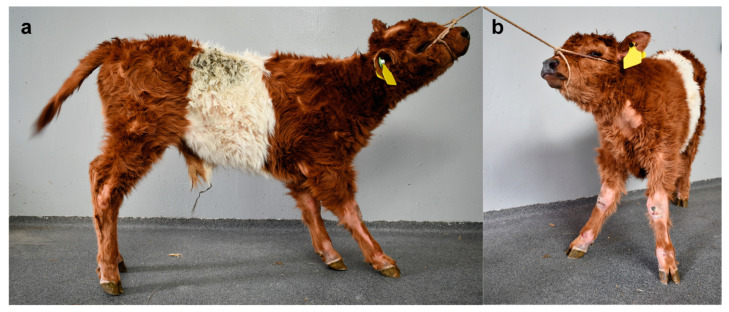
Congenital hypotrichosis in a Swiss Belted Galloway cattle. (**a**) Note the multiple areas of alopecia on the limbs and the dorsal part of the head. (**b**) Note the large alopecic lesions located on the neck and the dorsal aspect of the fetlock and carpal joints. Note also the excoriations on the dorsal aspect of the fetlock and carpal joints.

The integumentary system examination revealed multiple small to large areas of alopecia on the limbs and the dorsal part of the head, neck, and back (Figure 1a,b). The largest alopecic lesions were located on the lateral and medial aspects of the tarsal joints and the dorsal aspect of the fetlock and carpal joints. Moderate scaling was also present on the dorsal aspect of the head and neck. Excoriations were also present on the dorsal aspect of the fetlock and carpal joints and the lateral aspect of the tarsal joints. There was no evidence of erythema, pruritus, crusting, or thickening of the skin.

Complete blood count revealed mild erythrocytosis, hypochromia, thrombocytosis, and leukocytosis due to mature neutrophilia and monocytosis. Plasma biochemistry revealed mild hyperbilirubinemia, hypercalcemia, hyperphosphatemia, severely increased GGT activity, mildly increased GLDH and SDH activities, and mildly decreased BUN concentration. VBG analysis revealed mild hyperglycemia and hyperlactatemia (Table S2). The calf was confirmed to be negative for BVDV.

Considering the age of the calf, the distribution of skin lesions, and the absence of other clinically relevant clinical abnormalities, congenital non-syndromic hypotrichosis was suspected.

### 3.2. Genetic Analysis

**Figure 2:**
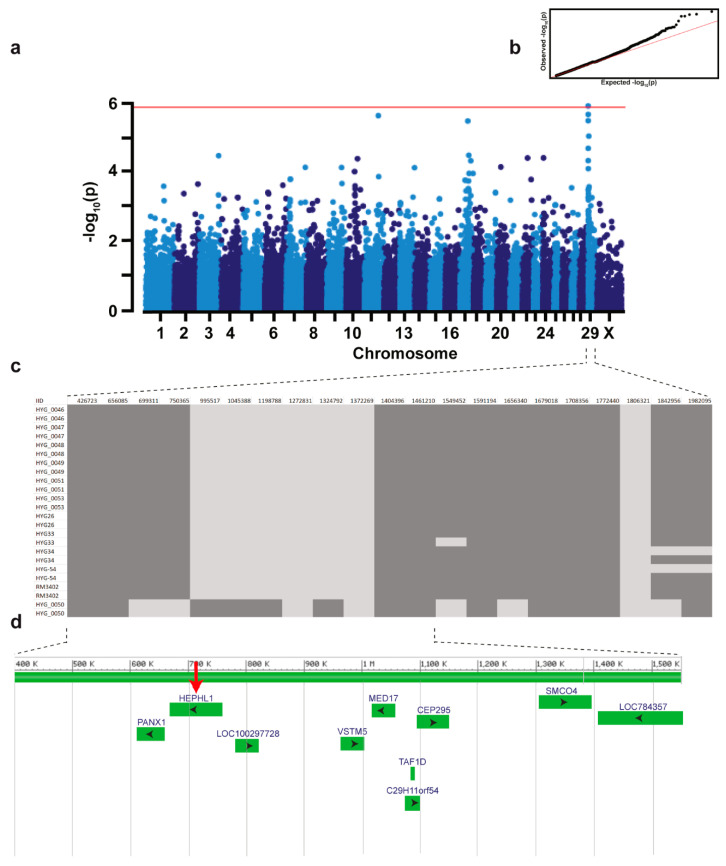
A region on chromosome 29, including the *HEPHL1* gene, is associated with recessive congenital hypotrichosis in Belted Galloway cattle. (**a**) Manhattan plot for the GWAS using 12 HY-suspected calves and 61 control cattle is shown and indicates a signal with multiple associated SNPs on chromosome 29. Each chromosome is represented along the X-axis. The Y-axis represents the −log_10_ of the corrected empirical *p*-value. The red line indicates the Bonferroni-corrected significance with a −log_10_*p* = 5.88 (*α* = 0.05). (**b**) The quantile-quantile plots showing the observed vs. expected log *p*-values are shown. (**c**) Schematic representing the haplotypes of 12 HY-suspected calves on chromosome 29. The exact positions of the SNP markers are indicated above. (**d**) Gene content of the 1.5 Mb-sized critical regions located at the proximal end of the chromosome. Screenshot of NCBI Genome Data Viewer (ARS-UCD1.2 assembly, *Bos taurus* annotation release 106) shows the annotated genes and loci including *HEPHL1* (red arrow).

A monogenic recessive mode of inheritance was hypothesized. GWAS using 12 cases and 61 controls revealed a region of genome-wide significance for association with the HY phenotype on chromosome 29 (Figure 2a). The best-associated SNP marker maps to position 1,045,388 bp (p_corrected_ = 1.22  ×  10^−6^). Haplotype analysis on this location revealed one region encompassing 21 markers of shared homozygosity in 11 affected calves located at the top of chromosome 29, with exception of one calf (case HYG_0050) (Figure 2c). It could be assumed that this single HY-suspected case was misclassified based on phenotype and was not affected with the same form of HY and therefore was considered to represent most likely a phenocopy. Recombinant haplotypes present in three cases (HYG33, HYG34, and HYG-54) allowed us to narrow the critical region for the HY-associated locus to a 1549-kb segment (Figure 2c).

**Figure 3:**
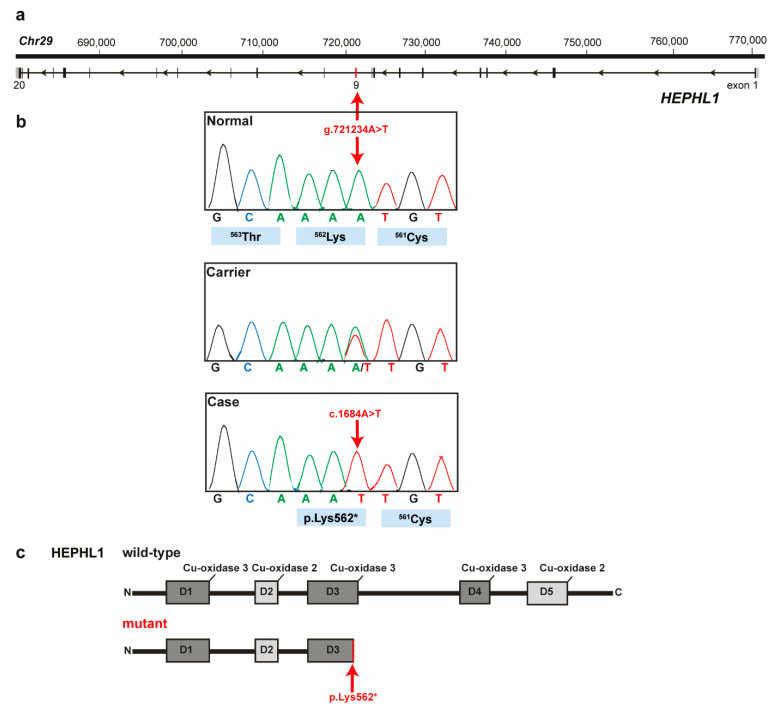
*HEPHL1* nonsense variant in Belted Galloway cattle affected by congenital hypotrichosis. (**a**) *HEPHL1* gene structure showing the location of the exon 9 variant on cattle chromosome 29. (**b**) Electropherograms showing the different genotypes identified via Sanger sequencing. (**c**) Schematic representation of HEPHL1 wild-type protein with its functional domains and the mutant protein. The wild-type protein is 1157 amino acid residues in length. The mutant protein is expected to be 562 amino acid residues in length due to the premature induction of a stop codon. D, domain.

Positional candidate genes within the 1549-kb critical region were selected based on function and location relative to the homozygosity analysis of the HY-affected calves. The coding exons and flanking splice regions of three positional candidate genes were subsequently re-sequenced: *CEP295*, *C29H11orf54*, and *HEPHL1*. Only variants that were predicted to alter the coding sequences or that were located within the splice sites were considered. A nonsense pathogenic variant was found in the ninth exon of the *HEPHL1* gene (chr29: g.721234A>T) (Figure 3a) which was consistent with the expected genotype status of the animals sequenced. At the level of translation, the detected single nucleotide substitution (c.1684A>T) is predicted to result in a premature stop codon in the Domain 3 (Cu-oxidase type 3) of HEPHL1 after lysine 562 (p.Lys562*) (Figure 3b,c). Consequently, the mutant protein, if expressed, is predicted to be significantly shorter than the wild-type HEPHL1 protein of 1157 amino acids in length lacking the Domains 4 (Cu-oxidase type 3) and 5 (Cu-oxidase type 2), and C-terminal domain. The HY protein is expected to be 562 amino acid residues in length due to the premature induction of a stop codon.

The nonsense variant in *HEPHL1* was further validated by Sanger sequencing (Figure 3b). Genotyping of all animals of the mapping cohort including the HY-calves and controls originating from the US Belted Galloway population plus the case and obligatory carrier (dam) and controls from Swiss herds, revealed almost perfect concordance between the presence of this nonsense variant and the HY phenotype (Table 1). As expected, 11 HY-affected calves were homozygous mutants and the available obligatory carriers were heterozygous. A single HY-suspected calf (case HyG_0050) did not show the deleterious variant in *HEPHL1*. It is highly probable that this calf was misclassified based on phenotype and was not affected with the same type of congenital hypotrichosis and therefore, as speculated before during haplotype analysis, indeed represent a phenocopy. By genotyping of almost 700 animals the mutant allele frequency was estimated as 2.2% and 6.7% in the studied Swiss and US Belted Galloway cattle populations, respectively. The higher allele frequency in the US population is probably biased as many animals submitted for diagnostics were of suspect pedigree, thus increasing the allele frequency artificially. Additionally, the identified *HEPHL1* variant was absent in a global control cohort of 4110 cattle genomes of a variety of breeds included in the run 8 of the 1000 Bull Genomes Project [36].

## 4. Discussion

The identified nonsense variant in *HEPHL1* resulting in a premature stop codon and consequent early truncation of the protein affects a functionally important site of a candidate gene and thus represents the most likely pathogenic variant associated with the observed recessively inherited congenital hypotrichosis (HY) phenotype in Belted Galloway cattle. This breed-specific disorder has not been reported before and we found purebred Belted Galloway calves from two different continents indicating that the mutant allele occurs worldwide segregating at different frequencies. To the best of our knowledge, no pathogenic variant in the *HEPHL1* gene has been reported in domestic animal species. Therefore, this study in cattle provides the first example of a *HEPHL1*-related congenital hair disorder in domestic animals.

In fact, iron represents an essential element and constituent of important cellular proteins such as myoglobin, hemoglobin, flavoproteins, cytochromes, and various non-heme enzymes [38]. Multi-copper ferroxidases play a major role in maintaining iron homeostasis in humans and mice [39,40,41]. In particular, these proteins present an important role in oxidizing ferrous iron [Fe (II)], released from the cells, into ferric iron [Fe (III)], which is subsequently distributed by transferrin [42]. Ceruloplasmin (CP) and hephaestin (HEPH) are well-known ferroxidases that facilitate this reaction in different tissues while the hephaestin like 1 (HEPHL1), another member of the multicopper oxidase family have an uncertain role in iron transport [39]. In humans, recessively inherited biallelic *HEPHL1* variants are associated with a phenotype characterized by abnormal hair (pili torti and trichorrhexis nodosa), joint laxity, severe heat intolerance, and developmental delay (OMIM 261990) [39]. In mice, recessively inherited mutations in *HEPHL1* are responsible for the so-called “hephaestin like 1; curly whiskers” (*Hephl1*^cw^) and “hephaestin like 1; curly whiskers 2 Jackson” (*Hephl1*^cw−2J^) phenotypes that are associated with abnormal hair-follicle development and immunogenetic disorders [40]. Remarkably, *HEPHL1*-homozygous mutant Belted Galloway cattle, clinically presented exclusively hair abnormalities and a slight alteration on hepatic parameters, supporting an important role for the ferroxidase activity of HEPHL1 in hair development and suggesting a possible role in iron homeostasis. The hair abnormalities described herein in Galloway cattle are distinguishable from those of HY in Hereford cattle [43]. Moreover, most changes detected in hematological and biochemical variables (i.e., erythrocytosis, hypochromasia, thrombocytosis, leukocytosis, neutrophilia, monocytosis, increased GGT activity, decreased BUN concentration, hyperglycemia, hypercalcemia, and hyperphosphatemia) were deemed age-related as similar changes were demonstrated in healthy calves during the first months of life [44]. Leukocytosis, neutrophilia, monocytosis, thrombocytosis, hyperlactatemia, and hyperglycemia could also have resulted from the endogenous release of catecholamines and glucocorticoids in response to transport and handling. Hypochromasia could also have been secondary to iron or copper deficiency. However, in the absence of anemia and microcytosis, this was deemed less likely. An increase in GGT activity in association with a normal plasma protein concentration was consistent with an adequate colostrum intake and transfer of passive immunity. Increased GLDH and SDH activities were consistent with mild acute hepatocellular injury. In the absence of increased AST activity, a primary hepatic disease was deemed unlikely while hyperbilirubinemia was consistent with cholestasis. Interestingly, mutations in CP are associated with aceruloplasminemia in humans (OMIM604290) and mice leading to a decrease in iron export and an increase of iron retention in the liver [45,46]. Given the retrieved hepatic parameters of the homozygous mutant calf in this study, we hypothesize by the similarity that a deficiency in HEPHL1 may also lead to an alteration of iron transport in the liver.

The pathogenic variant herein identified was positioned in Domain 3, a Cu-oxidase type 3, of the HEPHL1 bovine protein. Taking into account that the identified pathogenic variant is a nonsense variant resulting in a significantly shorter mutant protein since it encodes an early stop codon, we speculate that the identified amino-acid substitution disturbs the protein. In fact, if the mutant mRNA transcript were to escape nonsense-mediated decay [47], even if this truncated protein was produced, it would lack Domains 4 and 5, and C-terminal membrane-spanning domain, and therefore it is not expected to contribute any membrane ferroxidase function. Therefore, it is very unlikely that the mutant protein, if expressed, fulfills any physiological function. Possibly nonsense-mediated decay selectively recognizes and degrades truncated transcripts. We were not able to get skin biopsies for studying either RNA expression e.g., by RT-PCR or HEPHL1 protein expression e.g., by immunofluorescence.

## 5. Conclusions

Rare non-lethal disorders such as HY in livestock are commonly not reported or are misdiagnosed when the animals show mild to moderate phenotype. However, these disorders affect negatively animal welfare due to the development of secondary infections. Additionally, molecular diagnosis is often not performed because of a lack of resources and diagnostic tools. Moreover, this study provides a DNA-based diagnostic test that allows selection against the identified pathogenic variant in the international Belted Galloway cattle population. This diagnostic test will permit the avoidance of risk matings by systematic genetic testing of potential breeding animals and in particular top sires used in artificial insemination. Investigation of this phenotype allowed a clinical and molecular genetic study, enabling for the first time the diagnosis of a *HEPHL1*-related recessively inherited form of HY in domestic animals. This study represents the first large animal model for similar human conditions. Furthermore, this example highlights the utility of precision diagnostics for understanding rare disorders and the neglected value of livestock populations for studying genetic disorders.

## Figures and Tables

**Table 1:** Association of the nonsense variant in *HEPHL1* with the hypotrichosis phenotype in Belted Galloway cattle.

	TT	AT	AA
**HY-affected calves**			
Swiss case			1
US cases	1 ^a^		10
**Obligate carriers ^b^**			
Swiss		1	
US		18	
**Unrelated normal Belted Galloway cattle**			
Swiss	148	7	
US	471	73	
**Normal control cattle from various breeds**	4110		

^a^ this animal was assumed to represent a phenocopy (see main text) ^b^ these parents of HY-affected animals were classified as obligate carriers.

## Data Availability

Not applicable.

## Supplementary Materials

The following are available online at https://www.mdpi.com/article/10.3390/genes12050643/s1, Table S1: Primer Sequences. Table S2: Blood analysis results of a Swiss Belted Galloway calf affected by congenital hypotrichosis.
